# Ethyl (*E*)-3-[1′-ethyl-2-oxo-4′-(phenyl­sulfon­yl)-2*H*-spiro­[acenaphthyl­ene-1,2′-pyrrolidine]-3′-yl]acrylate

**DOI:** 10.1107/S1600536812016042

**Published:** 2012-04-18

**Authors:** Ulaganathan Sankar, R. Uma, S. Sundaramoorthy, D. Velmurugan

**Affiliations:** a Department of Chemistry, Pachaiyappas College, Chennai 600 030, India; b Centre of Advanced Study in Crystallography and Biophysics, University of Madras, Guindy Campus, Chennai 600 025, India

## Abstract

In the title compound, C_28_H_27_NO_5_S, the five-membered pyrrolidine ring, which exhibits an envelope conformation (the C atom at the spiral junction being the flap atom), makes dihedral angles of 57.37 (10) and 86.84 (8)°, respectively, with the phenyl ring and the acenaphthyl­ene ring system. In the crystal, mol­ecules associate *via* two C—H⋯O hydrogen bonds, forming *R*_2_^2^(20) and *R*_2_^2^(10) graph-set motifs.

## Related literature

For the biological activity of spiro compounds, see: Kobayashi *et al.* (1991 ▶); James *et al.* (1991 ▶); Obniska *et al.* (2003 ▶); Peddi *et al.* (2004 ▶). For ring conformational analysis, see: Cremer & Pople (1975 ▶).
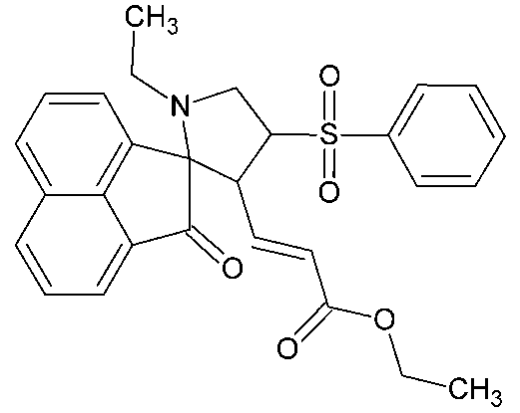


## Experimental

### 

#### Crystal data

C_28_H_27_NO_5_S
*M* *_r_* = 489.58
Triclinic, 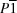

*a* = 11.1917 (8) Å
*b* = 11.7778 (9) Å
*c* = 12.1511 (9) Å
α = 93.572 (3)°
β = 115.911 (3)°
γ = 114.134 (3)°
*V* = 1256.82 (17) Å^3^
*Z* = 2
Mo *K*α radiation
μ = 0.17 mm^−1^
*T* = 295 K
0.26 × 0.23 × 0.20 mm

#### Data collection

Bruker SMART APEXII CCD diffractometer
Absorption correction: multi-scan (*SADABS*; Bruker, 2008 ▶) *T*_min_ = 0.957, *T*_max_ = 0.967
19294 measured reflections
6053 independent reflections
4834 reflections with *I* > 2σ(*I*)
*R*_int_ = 0.027

#### Refinement

*R*[*F* ^2^ > 2σ(*F* ^2^)] = 0.043
*wR*(*F* ^2^) = 0.127
*S* = 1.04
6053 reflections
318 parameters
H-atom parameters constrained
Δρ_max_ = 0.50 e Å^−3^
Δρ_min_ = −0.30 e Å^−3^

### 

Data collection: *APEX2* (Bruker, 2008 ▶); cell refinement: *SAINT* (Bruker, 2008 ▶); data reduction: *SAINT*; program(s) used to solve structure: *SHELXS97* (Sheldrick, 2008 ▶); program(s) used to refine structure: *SHELXL97* (Sheldrick, 2008 ▶); molecular graphics: *ORTEP-3* (Farrugia, 1997 ▶); software used to prepare material for publication: *SHELXL97* and *PLATON* (Spek, 2009 ▶).

## Supplementary Material

Crystal structure: contains datablock(s) global, I. DOI: 10.1107/S1600536812016042/rk2349sup1.cif

Structure factors: contains datablock(s) I. DOI: 10.1107/S1600536812016042/rk2349Isup2.hkl

Supplementary material file. DOI: 10.1107/S1600536812016042/rk2349Isup3.cml

Additional supplementary materials:  crystallographic information; 3D view; checkCIF report

## Figures and Tables

**Table 1:** Hydrogen-bond geometry (Å, °)

*D*—H⋯*A*	*D*—H	H⋯*A*	*D*⋯*A*	*D*—H⋯*A*
C21—H21*A*⋯O1^i^	0.97	2.53	3.452 (3)	160
C28—H28⋯O5^ii^	0.93	2.55	3.336 (3)	142

Symmetry codes: (i) 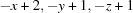; (ii) 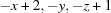.

**Fig. 1.**
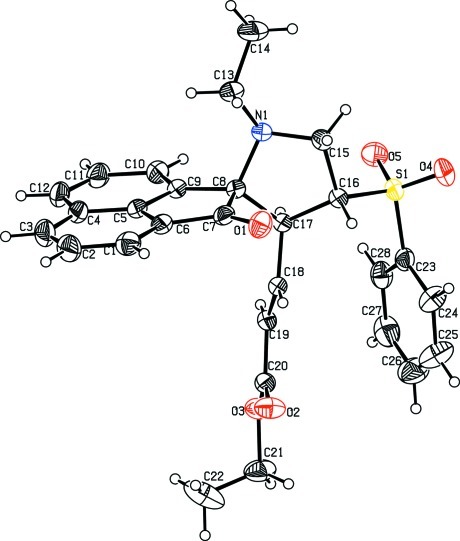
Perspective view of the molecule showing the atom numbering scheme. Displacement ellipsoids are drawn at 30% probability level. H atoms are presented as a small spheres of arbitrary radius.

**Fig. 2.**
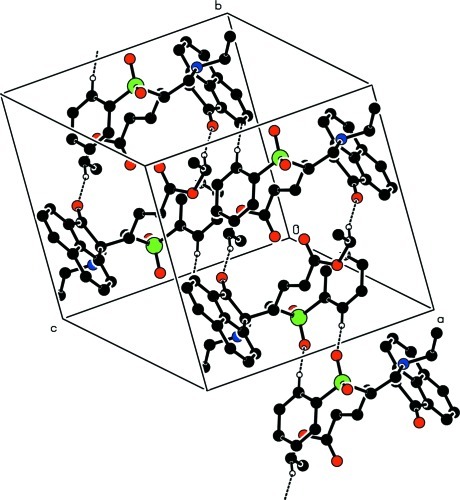
The crystal packing of the molecules viewed along the a–axis; H–bonds are shown as dashed lines forms R22(20) and R22(10) graph set motifs. For the sake of clarity, H atoms, not involved in hydrogen bonds, have been omitted.

## supplementary crystallographic information

### Comment

Spiro compounds are a particular class of naturally occurring substances characterized by highly pronounced biological properties (Kobayashi *et al.*, 1991; James *et al.*, 1991). Spiro-pyrrolidine derivatives are unique tetracyclic 5-HT(2 A) receptor antagonist (Obniska *et al.*, 2003; Peddi *et al.*, 2004). In view of this importance, the crystal structure of the title compound, has been determined and the results are presented here.

The pyrrolidine ring makes dihedral angles of 57.37 (10)° and 86.84 (8)° with the phenyl ring and the acenaphthylene ring system, respectively. The sum of the angles at N1 of the pyrrolidine ring - 335.5 (3)°) is in accordance with *sp*^3^ hybridization. The pyrrolidine ring adopts an envelope conformation with C8 deviating from the plane defined by the rest of the atoms of the ring by -0.2603 (2)Å. The puckering parameters (Cremer & Pople, 1975) of this ring are Q_T_= 0.4137 (2)Å and φ_2_ = 30.3 (3)°.

The crystal packing is stabilized by C—H···O intermolecular interactions. Atom C21 acts as a donor to O1^i^ and atom C28 acts as a donor to O5^ii^, generating *R_2_^2^*(20) and *R_2_^2^*(10) graph set motifs, respectively. Symmetry codes: (i) -*x*+2, -*y*+1, -*z*+1; (ii) -*x*+2, -*y*, -*z*+1.

### Experimental

The mixture of (2*E*,4*E*)–ethyl–5–(phenyl–sulfonyl)penta–2,4–dienoate (1 g, 3.8 mmol), *N*–ethyl glycine (0.43 g 4.1 mmol) and acenapthoquinone (0.75 g 4.1 mmol) in dry 1,4–dioxane (20 ml) was refluxed for 5 hr under N2 atm. The reaction mixture was concentreated to remove the solvent and then purified by column chromatography to yield the required title compound as a coloureless solid. The solvent accessible void 47Å^3^ is found in crystal.

### Refinement

The C bound H atoms positioned geometrically (C—H=0.93–0.98 Å) and allowed to ride on their parent atoms, with 1.5*U*_eq_(C) for methyl H and 1.2 *U*_eq_(C) for other H atoms.

### Crystal data

**Table:**

C_28_H_27_NO_5_S	*Z* = 2
*M**_r_* = 489.58	*F*(000) = 516
Triclinic, *P*1	*D*_x_ = 1.294 Mg m^−^^3^
Hall symbol: -P 1	Mo *K*α radiation, λ = 0.71073 Å
*a* = 11.1917 (8) Å	Cell parameters from 1225 reflections
*b* = 11.7778 (9) Å	θ = 2.0–28.3°
*c* = 12.1511 (9) Å	µ = 0.17 mm^−^^1^
α = 93.572 (3)°	*T* = 295 K
β = 115.911 (3)°	Block, colourless
γ = 114.134 (3)°	0.26 × 0.23 × 0.20 mm
*V* = 1256.82 (17) Å^3^	

### Data collection

**Table:**

Bruker SMART APEXII CCD diffractometer	6053 independent reflections
Radiation source: fine–focus sealed tube	4834 reflections with *I* > 2σ(*I*)
Graphite monochromator	*R*_int_ = 0.027
ω and φ scans	θ_max_ = 28.3°, θ_min_ = 2.0°
Absorption correction: multi-scan (*SADABS*; Bruker, 2008)	*h* = −14→14
*T*_min_ = 0.957, *T*_max_ = 0.967	*k* = −13→15
19294 measured reflections	*l* = −16→16

### Refinement

**Table:**

Refinement on *F*^2^	Primary atom site location: structure-invariant direct methods
Least-squares matrix: full	Secondary atom site location: difference Fourier map
*R*[*F*^2^ > 2σ(*F*^2^)] = 0.043	Hydrogen site location: inferred from neighbouring sites
*wR*(*F*^2^) = 0.127	H-atom parameters constrained
*S* = 1.04	*w* = 1/[σ^2^(*F*_o_^2^) + (0.0631*P*)^2^ + 0.2849*P*] where *P* = (*F*_o_^2^ + 2*F*_c_^2^)/3
6053 reflections	(Δ/σ)_max_ < 0.001
318 parameters	Δρ_max_ = 0.50 e Å^−^^3^
0 restraints	Δρ_min_ = −0.30 e Å^−^^3^

### Special details

**Table:**

Geometry. All s.u.'s (except the s.u. in the dihedral angle between two l.s. planes) are estimated using the full covariance matrix. The cell s.u.'s are taken into account individually in the estimation of s.u.'s in distances, angles and torsion angles; correlations between s.u.'s in cell parameters are only used when they are defined by crystal symmetry. An approximate (isotropic) treatment of cell s.u.'s is used for estimating s.u.'s involving l.s. planes.
Refinement. Refinement of *F*^2^ against ALL reflections. The weighted *R*–factor *wR* and goodness of fit *S* are based on *F*^2^, conventional *R*–factors *R* are based on *F*, with *F* set to zero for negative *F*^2^. The threshold expression of *F*^2^ > σ(*F*^2^) is used only for calculating *R*–factors(gt) *etc*. and is not relevant to the choice o f reflections for refinement. *R*–factors based on *F*^2^ are statistically about twice as large as those based on *F*, and *R*–factors based on ALL data will be even larger.

### Fractional atomic coordinates and isotropic or equivalent isotropic displacement parameters (Å^2^)

**Table:**

	*x*	*y*	*z*	*U*_iso_*/*U*_eq_	
C1	1.2133 (2)	0.56954 (17)	1.03581 (16)	0.0564 (4)	
H1	1.1535	0.6095	1.0209	0.068*	
C2	1.3587 (2)	0.6248 (2)	1.14553 (18)	0.0677 (5)	
H2	1.3955	0.7031	1.2030	0.081*	
C3	1.4474 (2)	0.5662 (2)	1.16999 (17)	0.0661 (5)	
H3	1.5428	0.6057	1.2438	0.079*	
C4	1.39774 (18)	0.44698 (17)	1.08589 (14)	0.0515 (4)	
C5	1.25279 (16)	0.39499 (15)	0.97617 (13)	0.0423 (3)	
C6	1.16211 (17)	0.45494 (15)	0.95136 (14)	0.0441 (3)	
C7	1.02002 (16)	0.37311 (15)	0.82823 (14)	0.0420 (3)	
C8	1.02678 (15)	0.24970 (14)	0.78074 (13)	0.0385 (3)	
C9	1.18392 (16)	0.27742 (14)	0.88182 (13)	0.0410 (3)	
C10	1.26179 (18)	0.21055 (17)	0.89651 (16)	0.0515 (4)	
H10	1.2195	0.1328	0.8356	0.062*	
C11	1.4085 (2)	0.2614 (2)	1.00628 (18)	0.0601 (4)	
H11	1.4614	0.2155	1.0160	0.072*	
C12	1.47483 (19)	0.3748 (2)	1.09791 (16)	0.0596 (5)	
H12	1.5711	0.4047	1.1685	0.071*	
C13	0.8902 (2)	0.12685 (18)	0.88435 (17)	0.0578 (4)	
H13A	0.8506	0.1842	0.8929	0.069*	
H13B	0.9899	0.1599	0.9595	0.069*	
C14	0.7862 (3)	−0.0082 (2)	0.8781 (2)	0.0868 (7)	
H14A	0.6855	−0.0386	0.8075	0.130*	
H14B	0.7842	−0.0057	0.9564	0.130*	
H14C	0.8230	−0.0660	0.8664	0.130*	
C15	0.76449 (17)	0.09965 (17)	0.65328 (15)	0.0534 (4)	
H15A	0.7127	0.1405	0.6712	0.064*	
H15B	0.6962	0.0066	0.6163	0.064*	
C16	0.81788 (15)	0.15707 (14)	0.56245 (13)	0.0407 (3)	
H16	0.7847	0.2215	0.5388	0.049*	
C17	0.99379 (15)	0.22744 (13)	0.64103 (13)	0.0368 (3)	
H17	1.0283	0.1673	0.6250	0.044*	
C18	1.06771 (15)	0.34865 (13)	0.60997 (12)	0.0370 (3)	
H18	1.0383	0.4115	0.6131	0.044*	
C19	1.17266 (15)	0.37120 (14)	0.57825 (13)	0.0389 (3)	
H19	1.2063	0.3110	0.5778	0.047*	
C20	1.23705 (16)	0.49004 (15)	0.54361 (14)	0.0414 (3)	
C21	1.3948 (2)	0.5929 (2)	0.4584 (2)	0.0715 (6)	
H21A	1.3187	0.6174	0.4098	0.086*	
H21B	1.4236	0.5647	0.4018	0.086*	
C22	1.5286 (3)	0.7062 (2)	0.5650 (4)	0.1173 (11)	
H22A	1.4980	0.7406	0.6154	0.176*	
H22B	1.5749	0.7718	0.5319	0.176*	
H22C	1.6003	0.6803	0.6176	0.176*	
C23	0.81600 (17)	0.11501 (15)	0.33263 (14)	0.0450 (3)	
C24	0.7631 (2)	0.1943 (2)	0.27206 (18)	0.0645 (5)	
H24	0.6808	0.1973	0.2709	0.077*	
C25	0.8334 (3)	0.2684 (3)	0.2137 (2)	0.0886 (7)	
H25	0.7995	0.3228	0.1740	0.106*	
C26	0.9531 (3)	0.2626 (3)	0.2136 (2)	0.0865 (7)	
H26	1.0004	0.3135	0.1744	0.104*	
C27	1.0036 (2)	0.1820 (2)	0.2713 (2)	0.0739 (6)	
H27	1.0832	0.1769	0.2689	0.089*	
C28	0.9364 (2)	0.10824 (18)	0.33293 (17)	0.0564 (4)	
H28	0.9718	0.0551	0.3738	0.068*	
N1	0.90471 (14)	0.12794 (12)	0.77021 (12)	0.0458 (3)	
O1	0.91411 (13)	0.39290 (12)	0.77286 (11)	0.0570 (3)	
O2	1.20878 (15)	0.57760 (12)	0.54698 (14)	0.0612 (3)	
O3	1.33098 (13)	0.48649 (11)	0.50468 (12)	0.0549 (3)	
O4	0.57643 (13)	−0.01410 (13)	0.35199 (13)	0.0698 (4)	
O5	0.78909 (16)	−0.06088 (11)	0.45257 (13)	0.0680 (4)	
S1	0.73711 (4)	0.03210 (4)	0.41992 (4)	0.04772 (12)	

### Atomic displacement parameters (Å^2^)

**Table:**

	*U*^11^	*U*^22^	*U*^33^	*U*^12^	*U*^13^	*U*^23^
C1	0.0689 (11)	0.0555 (10)	0.0484 (9)	0.0283 (9)	0.0346 (8)	0.0137 (8)
C2	0.0720 (12)	0.0613 (11)	0.0463 (9)	0.0173 (10)	0.0278 (9)	−0.0002 (8)
C3	0.0547 (10)	0.0728 (12)	0.0385 (8)	0.0146 (9)	0.0155 (8)	0.0035 (8)
C4	0.0432 (8)	0.0612 (10)	0.0355 (7)	0.0160 (7)	0.0171 (6)	0.0156 (7)
C5	0.0405 (7)	0.0466 (8)	0.0346 (7)	0.0155 (6)	0.0196 (6)	0.0162 (6)
C6	0.0487 (8)	0.0464 (8)	0.0374 (7)	0.0201 (7)	0.0245 (6)	0.0152 (6)
C7	0.0440 (7)	0.0482 (8)	0.0390 (7)	0.0225 (7)	0.0242 (6)	0.0190 (6)
C8	0.0356 (7)	0.0390 (7)	0.0366 (7)	0.0151 (6)	0.0171 (6)	0.0151 (6)
C9	0.0385 (7)	0.0427 (7)	0.0364 (7)	0.0167 (6)	0.0171 (6)	0.0169 (6)
C10	0.0489 (8)	0.0492 (9)	0.0508 (9)	0.0242 (7)	0.0204 (7)	0.0176 (7)
C11	0.0498 (9)	0.0730 (12)	0.0603 (10)	0.0359 (9)	0.0230 (8)	0.0315 (10)
C12	0.0404 (8)	0.0778 (12)	0.0447 (9)	0.0239 (8)	0.0131 (7)	0.0228 (9)
C13	0.0596 (10)	0.0599 (10)	0.0485 (9)	0.0185 (8)	0.0319 (8)	0.0239 (8)
C14	0.0939 (16)	0.0752 (14)	0.0774 (14)	0.0158 (12)	0.0540 (13)	0.0389 (12)
C15	0.0374 (7)	0.0598 (10)	0.0474 (8)	0.0108 (7)	0.0207 (7)	0.0195 (8)
C16	0.0334 (6)	0.0406 (7)	0.0392 (7)	0.0133 (6)	0.0156 (6)	0.0131 (6)
C17	0.0333 (6)	0.0363 (7)	0.0355 (6)	0.0140 (5)	0.0158 (5)	0.0123 (5)
C18	0.0344 (6)	0.0368 (7)	0.0339 (6)	0.0145 (5)	0.0151 (5)	0.0122 (5)
C19	0.0332 (6)	0.0379 (7)	0.0386 (7)	0.0147 (6)	0.0152 (6)	0.0113 (6)
C20	0.0352 (7)	0.0451 (8)	0.0433 (7)	0.0182 (6)	0.0202 (6)	0.0161 (6)
C21	0.0734 (12)	0.0852 (14)	0.0947 (15)	0.0458 (11)	0.0623 (12)	0.0546 (13)
C22	0.0925 (19)	0.0644 (15)	0.186 (3)	0.0142 (14)	0.086 (2)	0.0329 (18)
C23	0.0461 (8)	0.0407 (8)	0.0350 (7)	0.0187 (6)	0.0134 (6)	0.0051 (6)
C24	0.0807 (13)	0.0763 (13)	0.0578 (10)	0.0510 (11)	0.0382 (10)	0.0317 (10)
C25	0.132 (2)	0.0989 (17)	0.0813 (15)	0.0738 (17)	0.0701 (16)	0.0562 (14)
C26	0.1058 (18)	0.0906 (16)	0.0697 (13)	0.0378 (15)	0.0579 (14)	0.0316 (12)
C27	0.0615 (11)	0.0879 (15)	0.0614 (11)	0.0273 (11)	0.0338 (10)	0.0014 (11)
C28	0.0532 (9)	0.0541 (10)	0.0500 (9)	0.0267 (8)	0.0178 (8)	0.0031 (8)
N1	0.0395 (6)	0.0447 (7)	0.0412 (6)	0.0105 (5)	0.0197 (5)	0.0180 (5)
O1	0.0546 (7)	0.0699 (8)	0.0529 (7)	0.0385 (6)	0.0244 (6)	0.0199 (6)
O2	0.0699 (8)	0.0539 (7)	0.0907 (9)	0.0373 (6)	0.0559 (7)	0.0374 (7)
O3	0.0505 (6)	0.0576 (7)	0.0732 (8)	0.0278 (5)	0.0415 (6)	0.0305 (6)
O4	0.0377 (6)	0.0700 (8)	0.0584 (7)	0.0078 (6)	0.0094 (5)	0.0079 (6)
O5	0.0770 (9)	0.0391 (6)	0.0711 (8)	0.0264 (6)	0.0260 (7)	0.0188 (6)
S1	0.0393 (2)	0.0385 (2)	0.0427 (2)	0.01109 (16)	0.01077 (16)	0.00891 (15)

### Geometric parameters (Å, º)

**Table:**

C1—C6	1.373 (2)	C15—H15B	0.9700
C1—C2	1.406 (3)	C16—C17	1.5488 (18)
C1—H1	0.9300	C16—S1	1.7914 (16)
C2—C3	1.371 (3)	C16—H16	0.9800
C2—H2	0.9300	C17—C18	1.4963 (18)
C3—C4	1.418 (3)	C17—H17	0.9800
C3—H3	0.9300	C18—C19	1.3236 (19)
C4—C5	1.407 (2)	C18—H18	0.9300
C4—C12	1.412 (3)	C19—C20	1.473 (2)
C5—C6	1.402 (2)	C19—H19	0.9300
C5—C9	1.408 (2)	C20—O2	1.1993 (19)
C6—C7	1.478 (2)	C20—O3	1.3411 (18)
C7—O1	1.2085 (18)	C21—O3	1.451 (2)
C7—C8	1.573 (2)	C21—C22	1.468 (4)
C8—N1	1.4703 (17)	C21—H21A	0.9700
C8—C9	1.5168 (19)	C21—H21B	0.9700
C8—C17	1.5506 (19)	C22—H22A	0.9600
C9—C10	1.362 (2)	C22—H22B	0.9600
C10—C11	1.420 (2)	C22—H22C	0.9600
C10—H10	0.9300	C23—C28	1.381 (2)
C11—C12	1.363 (3)	C23—C24	1.386 (2)
C11—H11	0.9300	C23—S1	1.7609 (16)
C12—H12	0.9300	C24—C25	1.373 (3)
C13—N1	1.465 (2)	C24—H24	0.9300
C13—C14	1.511 (3)	C25—C26	1.369 (4)
C13—H13A	0.9700	C25—H25	0.9300
C13—H13B	0.9700	C26—C27	1.373 (3)
C14—H14A	0.9600	C26—H26	0.9300
C14—H14B	0.9600	C27—C28	1.386 (3)
C14—H14C	0.9600	C27—H27	0.9300
C15—N1	1.469 (2)	C28—H28	0.9300
C15—C16	1.540 (2)	O4—S1	1.4345 (12)
C15—H15A	0.9700	O5—S1	1.4383 (13)
			
C6—C1—C2	118.18 (18)	C17—C16—S1	113.76 (10)
C6—C1—H1	120.9	C15—C16—H16	108.8
C2—C1—H1	120.9	C17—C16—H16	108.8
C3—C2—C1	121.68 (18)	S1—C16—H16	108.8
C3—C2—H2	119.2	C18—C17—C16	113.82 (11)
C1—C2—H2	119.2	C18—C17—C8	114.52 (11)
C2—C3—C4	121.80 (17)	C16—C17—C8	102.29 (10)
C2—C3—H3	119.1	C18—C17—H17	108.6
C4—C3—H3	119.1	C16—C17—H17	108.6
C5—C4—C12	116.31 (16)	C8—C17—H17	108.6
C5—C4—C3	115.20 (17)	C19—C18—C17	123.55 (13)
C12—C4—C3	128.50 (16)	C19—C18—H18	118.2
C6—C5—C4	122.94 (15)	C17—C18—H18	118.2
C6—C5—C9	113.52 (13)	C18—C19—C20	121.14 (14)
C4—C5—C9	123.54 (15)	C18—C19—H19	119.4
C1—C6—C5	120.19 (15)	C20—C19—H19	119.4
C1—C6—C7	132.45 (16)	O2—C20—O3	123.92 (14)
C5—C6—C7	107.36 (13)	O2—C20—C19	125.58 (13)
O1—C7—C6	127.61 (15)	O3—C20—C19	110.49 (13)
O1—C7—C8	124.66 (14)	O3—C21—C22	111.1 (2)
C6—C7—C8	107.71 (12)	O3—C21—H21A	109.4
N1—C8—C9	112.97 (11)	C22—C21—H21A	109.4
N1—C8—C17	101.47 (11)	O3—C21—H21B	109.4
C9—C8—C17	115.83 (11)	C22—C21—H21B	109.4
N1—C8—C7	112.47 (12)	H21A—C21—H21B	108.0
C9—C8—C7	102.36 (11)	C21—C22—H22A	109.5
C17—C8—C7	112.18 (11)	C21—C22—H22B	109.5
C10—C9—C5	118.51 (14)	H22A—C22—H22B	109.5
C10—C9—C8	132.53 (14)	C21—C22—H22C	109.5
C5—C9—C8	108.93 (13)	H22A—C22—H22C	109.5
C9—C10—C11	118.95 (16)	H22B—C22—H22C	109.5
C9—C10—H10	120.5	C28—C23—C24	120.76 (17)
C11—C10—H10	120.5	C28—C23—S1	120.03 (13)
C12—C11—C10	122.53 (17)	C24—C23—S1	119.04 (13)
C12—C11—H11	118.7	C25—C24—C23	119.33 (19)
C10—C11—H11	118.7	C25—C24—H24	120.3
C11—C12—C4	120.15 (15)	C23—C24—H24	120.3
C11—C12—H12	119.9	C26—C25—C24	120.4 (2)
C4—C12—H12	119.9	C26—C25—H25	119.8
N1—C13—C14	111.99 (16)	C24—C25—H25	119.8
N1—C13—H13A	109.2	C25—C26—C27	120.3 (2)
C14—C13—H13A	109.2	C25—C26—H26	119.8
N1—C13—H13B	109.2	C27—C26—H26	119.8
C14—C13—H13B	109.2	C26—C27—C28	120.3 (2)
H13A—C13—H13B	107.9	C26—C27—H27	119.8
C13—C14—H14A	109.5	C28—C27—H27	119.8
C13—C14—H14B	109.5	C23—C28—C27	118.84 (18)
H14A—C14—H14B	109.5	C23—C28—H28	120.6
C13—C14—H14C	109.5	C27—C28—H28	120.6
H14A—C14—H14C	109.5	C13—N1—C15	113.66 (13)
H14B—C14—H14C	109.5	C13—N1—C8	114.79 (13)
N1—C15—C16	104.64 (11)	C15—N1—C8	107.01 (11)
N1—C15—H15A	110.8	C20—O3—C21	116.87 (13)
C16—C15—H15A	110.8	O4—S1—O5	118.19 (8)
N1—C15—H15B	110.8	O4—S1—C23	109.19 (8)
C16—C15—H15B	110.8	O5—S1—C23	108.26 (8)
H15A—C15—H15B	108.9	O4—S1—C16	107.00 (8)
C15—C16—C17	105.89 (11)	O5—S1—C16	109.48 (8)
C15—C16—S1	110.67 (10)	C23—S1—C16	103.78 (7)
			
C6—C1—C2—C3	−1.0 (3)	S1—C16—C17—C8	−142.41 (10)
C1—C2—C3—C4	0.2 (3)	N1—C8—C17—C18	162.09 (12)
C2—C3—C4—C5	0.6 (3)	C9—C8—C17—C18	−75.18 (16)
C2—C3—C4—C12	−179.67 (18)	C7—C8—C17—C18	41.83 (16)
C12—C4—C5—C6	179.53 (14)	N1—C8—C17—C16	38.46 (13)
C3—C4—C5—C6	−0.7 (2)	C9—C8—C17—C16	161.19 (12)
C12—C4—C5—C9	0.4 (2)	C7—C8—C17—C16	−81.79 (13)
C3—C4—C5—C9	−179.81 (14)	C16—C17—C18—C19	−127.00 (14)
C2—C1—C6—C5	0.9 (2)	C8—C17—C18—C19	115.79 (15)
C2—C1—C6—C7	−178.66 (15)	C17—C18—C19—C20	177.52 (12)
C4—C5—C6—C1	0.0 (2)	C18—C19—C20—O2	4.1 (2)
C9—C5—C6—C1	179.14 (13)	C18—C19—C20—O3	−174.71 (13)
C4—C5—C6—C7	179.61 (13)	C28—C23—C24—C25	−1.1 (3)
C9—C5—C6—C7	−1.21 (16)	S1—C23—C24—C25	174.11 (17)
C1—C6—C7—O1	1.0 (3)	C23—C24—C25—C26	1.0 (4)
C5—C6—C7—O1	−178.59 (14)	C24—C25—C26—C27	0.4 (4)
C1—C6—C7—C8	−177.43 (16)	C25—C26—C27—C28	−1.7 (4)
C5—C6—C7—C8	2.98 (15)	C24—C23—C28—C27	−0.1 (3)
O1—C7—C8—N1	−60.44 (18)	S1—C23—C28—C27	−175.30 (13)
C6—C7—C8—N1	118.04 (12)	C26—C27—C28—C23	1.5 (3)
O1—C7—C8—C9	178.03 (14)	C14—C13—N1—C15	68.2 (2)
C6—C7—C8—C9	−3.49 (13)	C14—C13—N1—C8	−168.20 (17)
O1—C7—C8—C17	53.20 (18)	C16—C15—N1—C13	158.39 (14)
C6—C7—C8—C17	−128.32 (12)	C16—C15—N1—C8	30.61 (17)
C6—C5—C9—C10	−179.75 (13)	C9—C8—N1—C13	64.42 (18)
C4—C5—C9—C10	−0.6 (2)	C17—C8—N1—C13	−170.90 (13)
C6—C5—C9—C8	−1.16 (16)	C7—C8—N1—C13	−50.85 (17)
C4—C5—C9—C8	178.02 (13)	C9—C8—N1—C15	−168.47 (13)
N1—C8—C9—C10	59.9 (2)	C17—C8—N1—C15	−43.79 (15)
C17—C8—C9—C10	−56.5 (2)	C7—C8—N1—C15	76.27 (15)
C7—C8—C9—C10	−178.87 (16)	O2—C20—O3—C21	−3.2 (2)
N1—C8—C9—C5	−118.37 (13)	C19—C20—O3—C21	175.66 (14)
C17—C8—C9—C5	125.19 (13)	C22—C21—O3—C20	82.8 (2)
C7—C8—C9—C5	2.81 (14)	C28—C23—S1—O4	−145.29 (14)
C5—C9—C10—C11	0.3 (2)	C24—C23—S1—O4	39.47 (16)
C8—C9—C10—C11	−177.86 (15)	C28—C23—S1—O5	−15.38 (15)
C9—C10—C11—C12	0.0 (3)	C24—C23—S1—O5	169.37 (14)
C10—C11—C12—C4	−0.2 (3)	C28—C23—S1—C16	100.88 (14)
C5—C4—C12—C11	−0.1 (2)	C24—C23—S1—C16	−74.36 (15)
C3—C4—C12—C11	−179.79 (17)	C15—C16—S1—O4	66.64 (13)
N1—C15—C16—C17	−4.79 (17)	C17—C16—S1—O4	−174.29 (10)
N1—C15—C16—S1	118.93 (12)	C15—C16—S1—O5	−62.57 (13)
C15—C16—C17—C18	−144.74 (13)	C17—C16—S1—O5	56.50 (12)
S1—C16—C17—C18	93.50 (13)	C15—C16—S1—C23	−177.97 (11)
C15—C16—C17—C8	−20.65 (15)	C17—C16—S1—C23	−58.90 (12)

### Hydrogen-bond geometry (Å, º)

**Table:**

*D*—H···*A*	*D*—H	H···*A*	*D*···*A*	*D*—H···*A*
C21—H21*A*···O1^i^	0.97	2.53	3.452 (3)	160
C28—H28···O5^ii^	0.93	2.55	3.336 (3)	142

Symmetry codes: (i) −*x*+2, −*y*+1, −*z*+1; (ii) −*x*+2, −*y*, −*z*+1.
